# Supplementary figures and images for: Yap suppresses T-cell function and infiltration in the tumor microenvironment
Source: PLoS Biol. 2020 Jan 13;18(1):e3000591. doi: 10.1371/journal.pbio.3000591 (PMC6980695; doi:10.1371/journal.pbio.3000591)

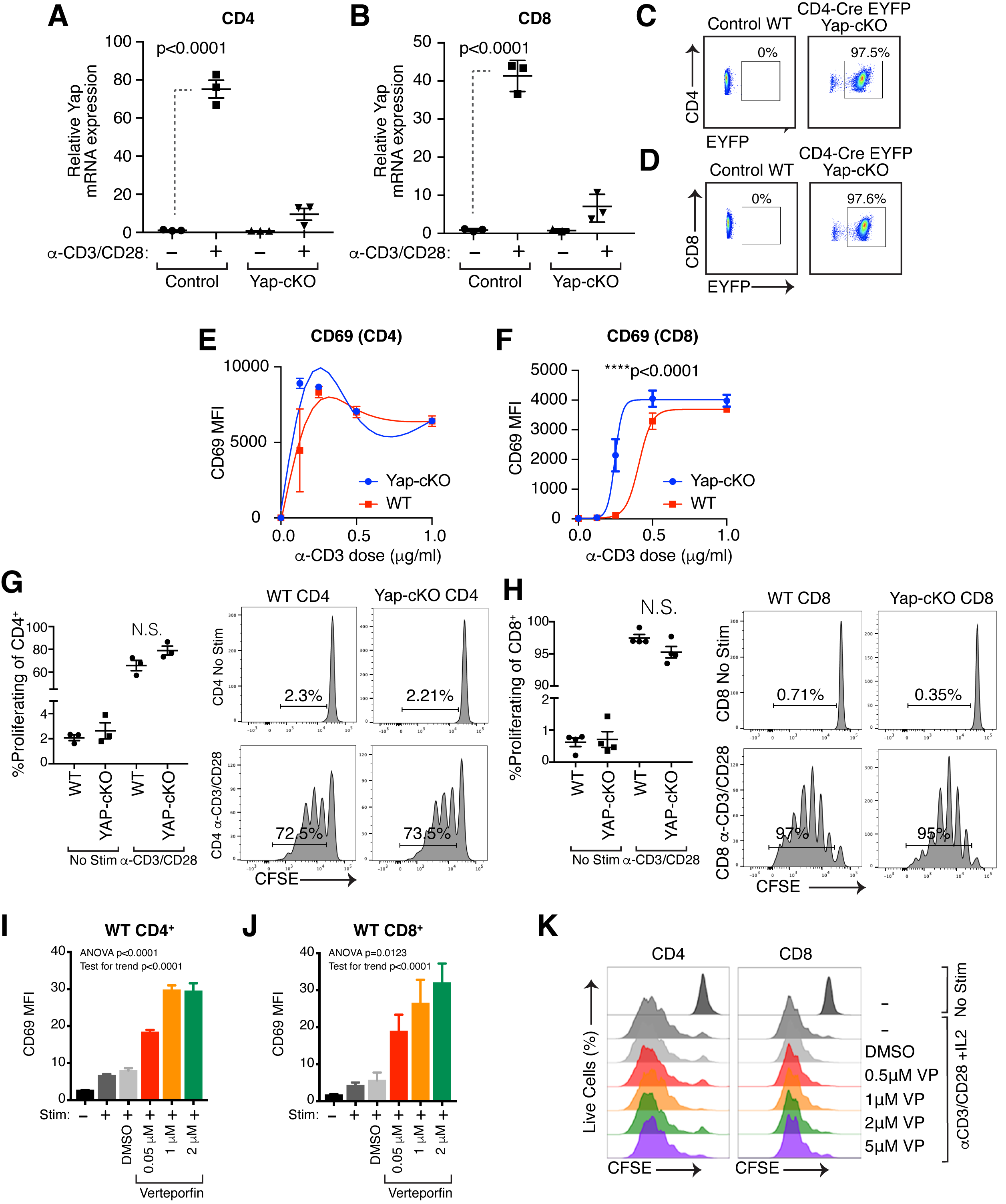

Supplement: S1 Fig — CD4+ and CD8+ T cells were isolated from WT or Yap-cKO mice and screened for EYFP expression as well as activation marker expression and proliferation after αCD3 and αCD28 stimulation. Proliferation was also tested for WT CD4+ and CD8+ T cells treated with increasing concentrations of verteporfin under IL-2, anti-CD3, and anti-CD28 stimulation. Statistical differences were determined by using a Student t test, with “ns” indicating not significant. (A) Yap mRNA expression in WT or Yap-cKO CD4+ T cells following CD3/CD28 stimulation. (B) Yap mRNA expression in WT or Yap-cKO CD8+ T cells following CD3/CD28 stimulation. (C) EYFP expression by flow cytometry on CD4+ cells isolated from WT or Yap-cKO mouse spleens. (D) EYFP expression by flow cytometry on CD8+ cells isolated from WT or Yap-cKO mouse spleens. (E) CD69 expression on WT and Yap-cKO CD4+ T cells 72 hours post CD3/CD28 stimulation (n = 2–3 per dose/group). (F) CD69 expression on WT and Yap-cKO CD8+ T cells 72 hours post CD3/CD28 stimulation (n = 2–3 per dose/group). (G) WT and Yap-cKO CD4+ T-cell proliferation (n = 3/group). (H) WT and Yap-cKO CD8+ T-cell proliferation (n = 3/group). (I) CD69 expression on WT CD4+ T cells 72 hours post IL-2 and CD3/CD28 stimulation and increasing concentration of verteporfin (n = 4/group). (J) CD69 expression on WT CD4+ T cells 72 hours post IL-2 and CD3/CD28 stimulation and increasing concentration of verteporfin (n = 4/group). (K) Proliferation of DMSO- versus verteporfin-treated WT CD4+ and CD8+ T cells (representative of 4 independent experiments). Raw data for this experiment are available in FLOWRepository (Repository ID: FR-FCM-Z2D5). (TIF) [file pbio.3000591.s001.tif]

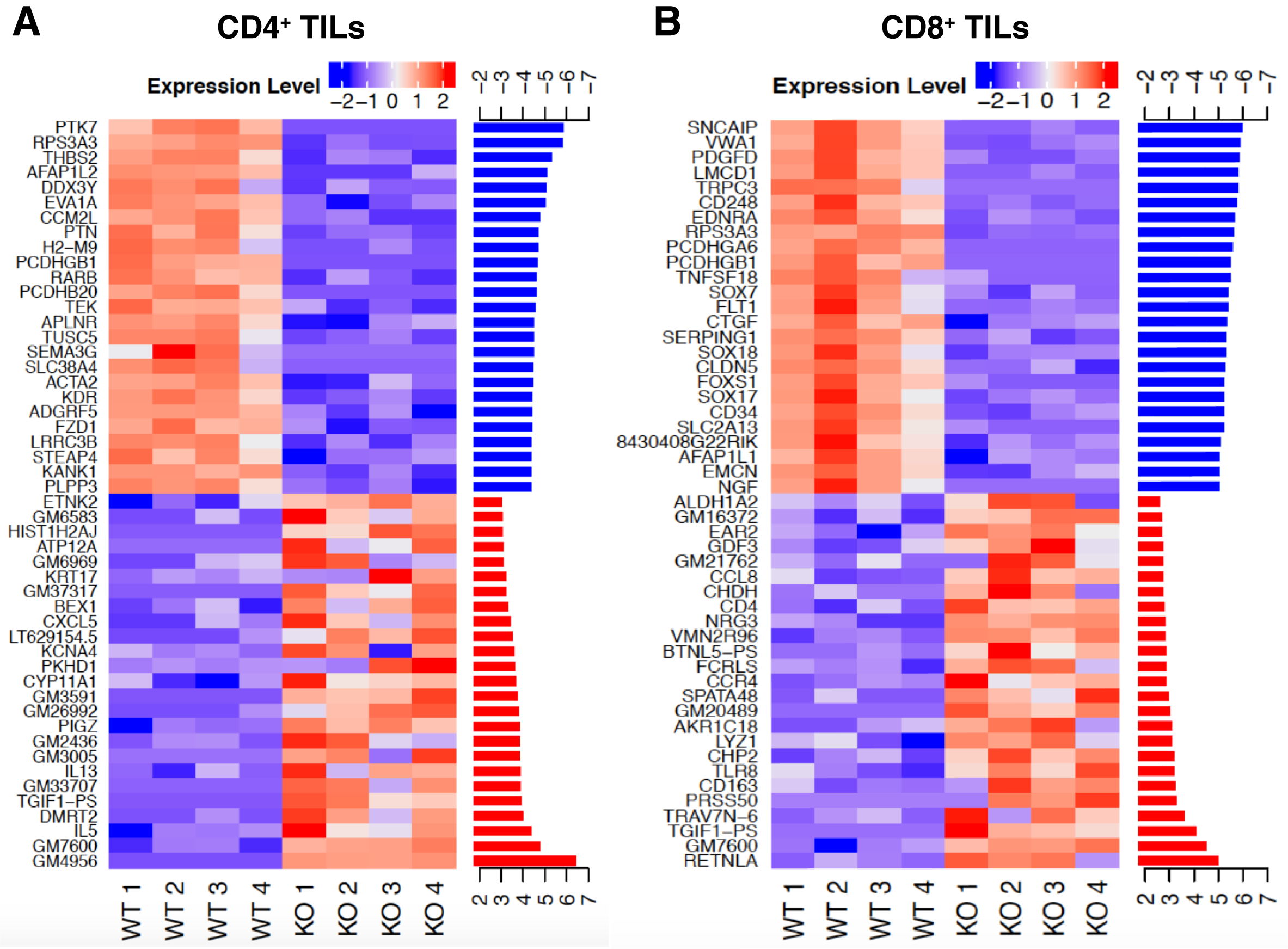

Supplement: S2 Fig — RNA-seq was performed from CD4+ and CD8+ TILs and TDLNs that were isolated from WT and Yap-cKO mice challenged with B16F10 tumors (data at NCBI GEO GSE139883 and listed in S1 and S2 Tables), and the top DEGs are shown. (A) A heatmap representing the top and bottom 25 DEGs in Yap-cKO versus WT CD4+ TILs. (B) A heatmap representing the top and bottom 25 DEGs in Yap-cKO versus WT CD8+ TILs. (TIF) [file pbio.3000591.s002.tif]

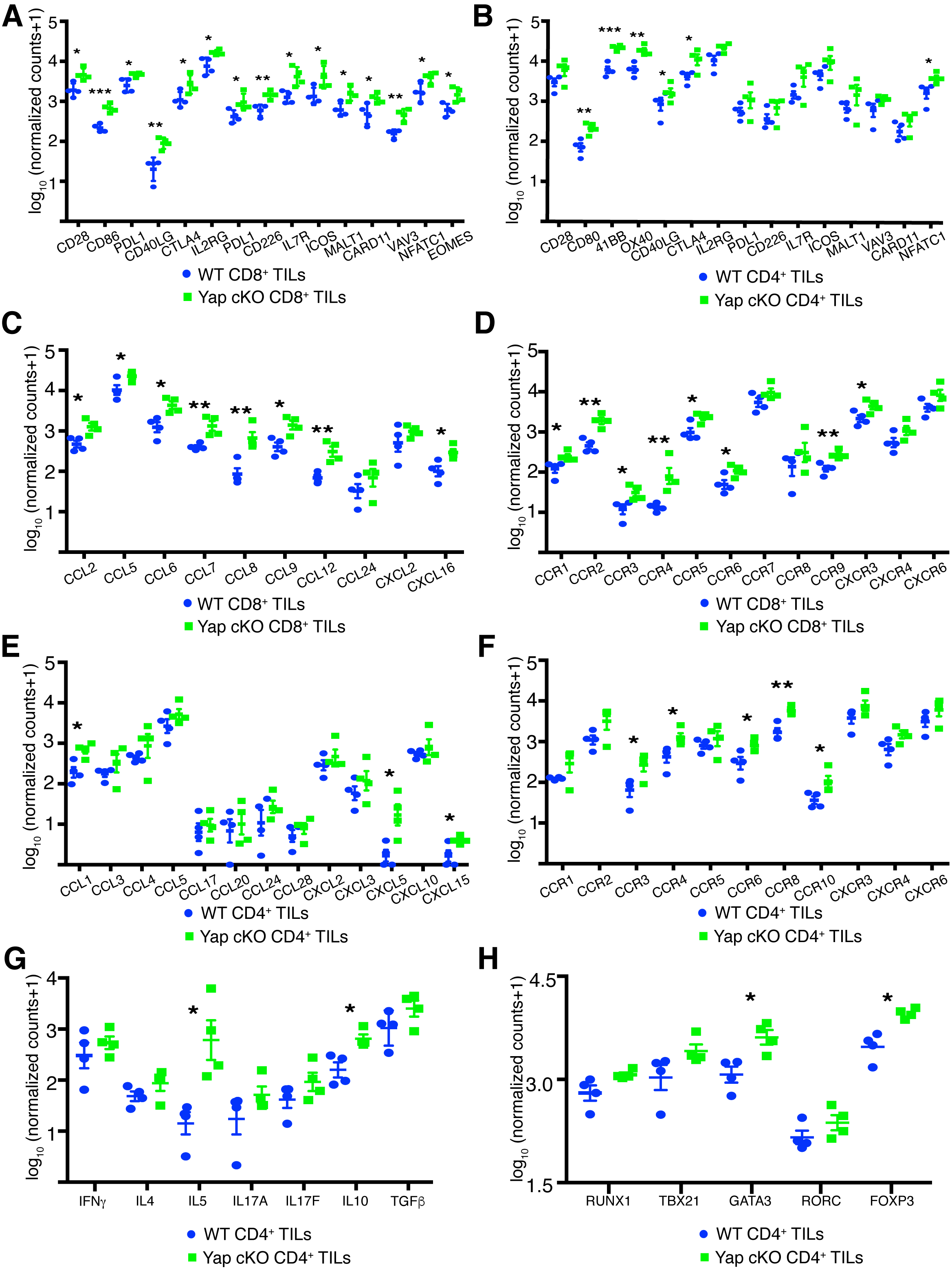

Supplement: S3 Fig — DEGs identified in Yap-cKO versus WT CD4+ and CD8+ TILs that encode factors related to T-cell function are shown. These data were derived from RNA-seq analysis of the respective mice challenged with B16F10 tumors, which is available at NCBI GEO (GSE139883) and listed in S1 and S2 Tables. (A) Log10(normalized RNA-seq counts +1) of T-cell activation–related genes in Yap-cKO versus WT CD8+ TILs. (B) Log10(normalized RNA-seq counts +1) of T-cell activation–related genes in Yap-cKO versus WT CD4+ TILs. (C) Log10(normalized RNA-seq counts +1) of chemokine genes in Yap-cKO versus WT CD8+ TILs. (D) Log10(normalized RNA-seq counts +1) of chemokine receptor genes in Yap-cKO versus WT CD8+ TILs. (E) Log10(normalized RNA-seq counts +1) of chemokine genes in Yap-cKO versus WT CD4+ TILs. (F) Log10(normalized RNA-seq counts +1) of chemokine receptor genes in Yap-cKO versus WT CD4+ TILs. (G) Log10(normalized RNA-seq counts +1) of T-helper subset–defining cytokines in Yap-cKO versus WT CD4+ TILs. (H) Log10(normalized RNA-seq counts +1) of T-helper subset–defining transcription factors in Yap-cKO versus WT CD4+ TILs. Significant differences were determined by a Student t test; *p < 0.05; **p < 0.01; ***p < 0.001. (TIF) [file pbio.3000591.s003.tif]

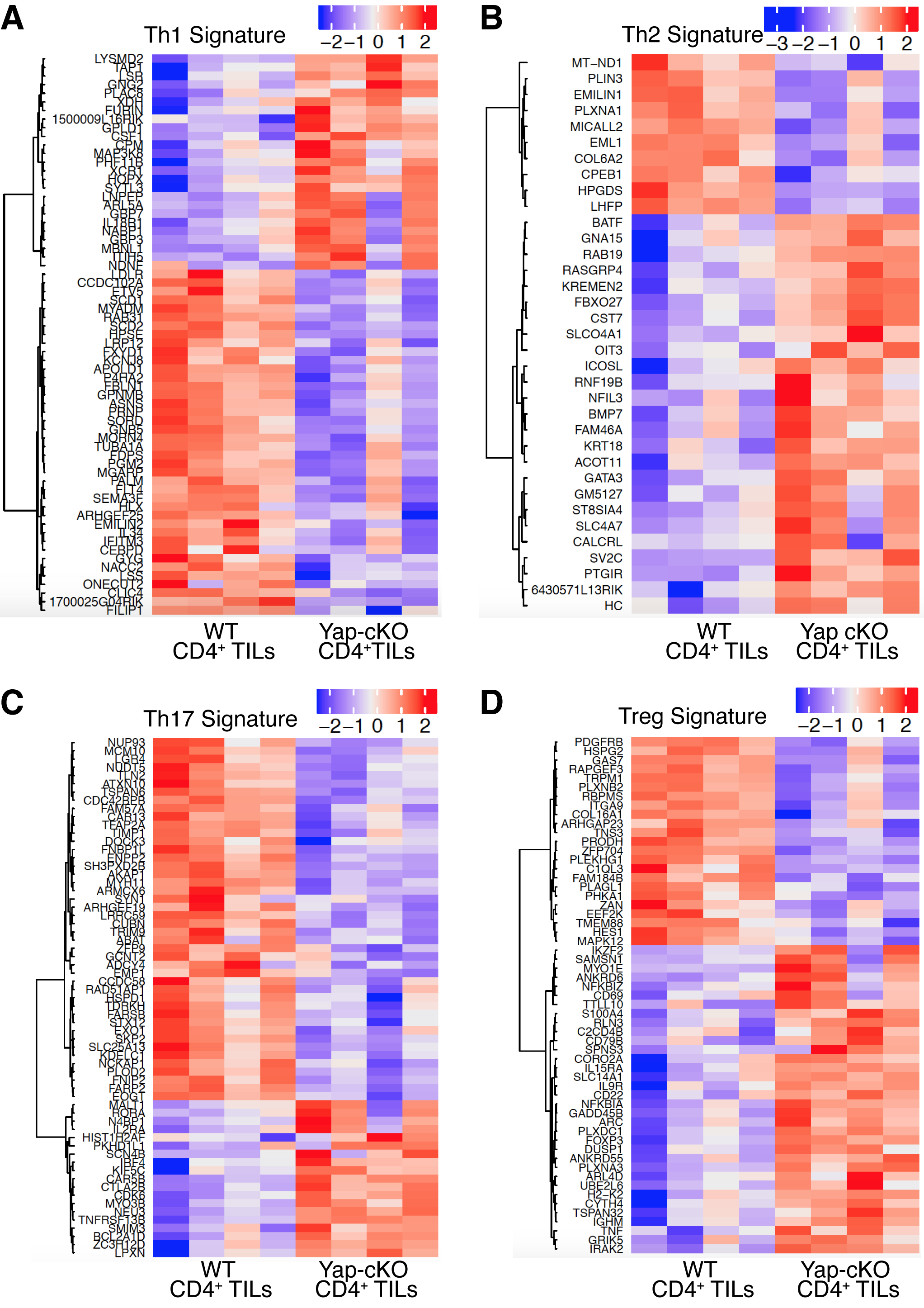

Supplement: S4 Fig — DEGs identified in Yap-cKO versus WT CD4+ TILs that represent different CD4+ fates are shown. These data were derived from RNA-seq analysis of the respective mice challenged with B16F10 tumors, which is available at NCBI GEO (GSE139883) and listed in S1 and S2 Tables. (A) Heatmap of statistically significant differentially expressed Th1-related genes in Yap-cKO versus WT CD4+ TILs. (B) Heatmap of statistically significant differentially expressed Th2-related genes in Yap-cKO versus WT CD4+ TILs. (C) Heatmap of statistically significant differentially expressed Th17-related genes in Yap-cKO versus WT CD4+ TILs. (D) Heatmap of statistically significant differentially expressed Treg-related genes in Yap-cKO versus WT CD4+ TILs. (TIF) [file pbio.3000591.s004.tif]

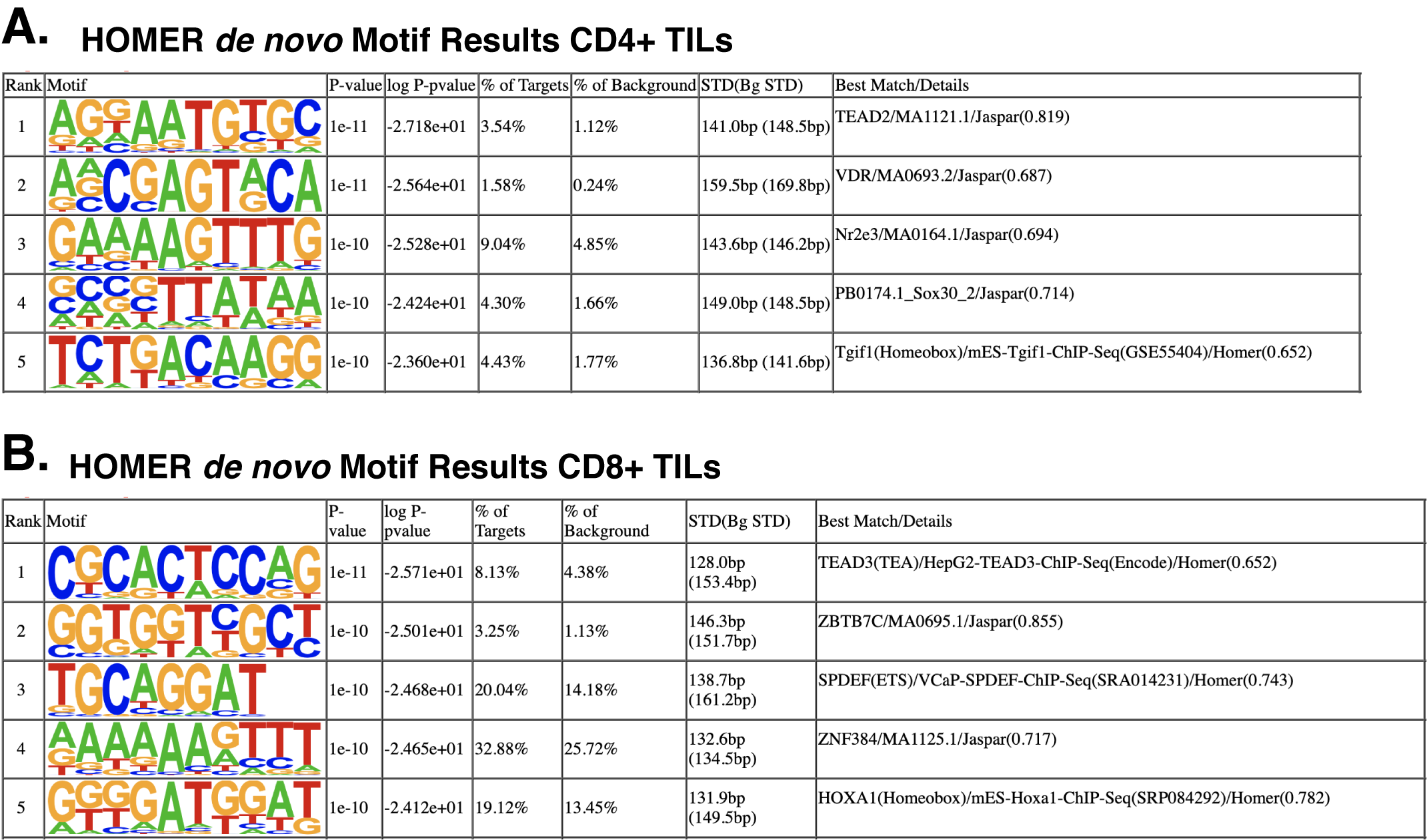

Supplement: S5 Fig — HOMER de novo motif analysis was performed on down-regulated gene expression changes identified in Yap-cKO versus WT (A) CD4+ and (B) CD8+ TILs, revealing the TEAD transcription factor motifs among the top enriched motifs. (TIF) [file pbio.3000591.s005.tif]
