# Supplementary material for: Yap suppresses T-cell function and infiltration in the tumor microenvironment
Source: PLoS Biol. 2020 Jan 13;18(1):e3000591. doi: 10.1371/journal.pbio.3000591 (PMC6980695; doi:10.1371/journal.pbio.3000591)
Supplement: S1 Raw Images — Yap immunoblots from (A) CD4+ and (B) CD8+ cells, and GAPDH immunoblots from (C) CD4+ and (D) CD8+ cells. (PDF) [file pbio.3000591.s015.pdf]

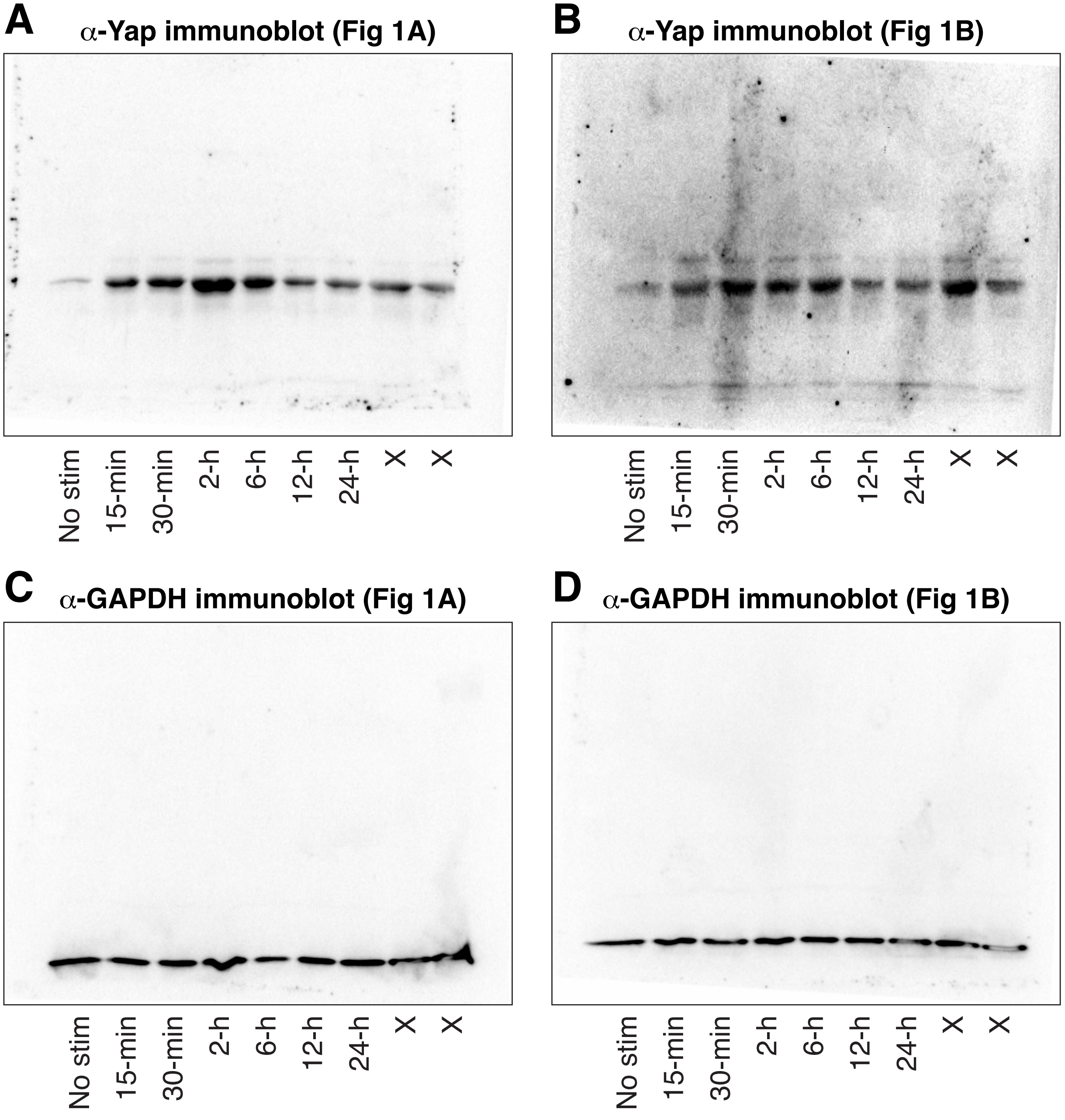

Original Immunoblot images from Fig 1A-B were captured using a Bio-Rad ChemiDoc system.
