# Supplementary material for: Yap suppresses T-cell function and infiltration in the tumor microenvironment
Source: PLoS Biol. 2020 Jan 13;18(1):e3000591. doi: 10.1371/journal.pbio.3000591 (PMC6980695; doi:10.1371/journal.pbio.3000591)
Supplement: Attachment — Submitted filename: Stampouloglou_Response.docx [file pbio.3000591.s016.docx]

**Response to the reviewers’ comments**

**Reviewer #1:** This is an interesting and very well performed study that investigates the role of the central Hippo pathway effector Yap in different T cell subsets. The role of this pathway in blood is underappreciated and, as such, the manuscript is valuable as it reveals new insights into how it functions in T cells. A previous study in Cancer Discovery had reported a role for YAP in T cell immunity and specifically that it was required for Treg differentiation. The present study is distinct from this as they define previously unappreciated roles for Yap in CD8+ T cells and other T cell subsets in the context of cancer immunity. The find that Yap expression is elevated in activated T cells and that Yap deletion causes elevated T-cell activation and differentiation. This gives new insights into the normal role of Hippo/Yap in the immune compartment and, given the authors’ findings that tumor growth is repressed in their Yap KO T cell models, further rationale for the use of YAP inhibitors in human cancers. Mechanistically, they show that Yap-deficient T cells have a greater ability to infiltrate tumors and they explore this further using RNA-seq - again, this is very relevant for the burgeoning field of cancer immunotherapy. They also explore TCGA datasets with their YAP signatures and find correlations with patient outcomes in several different cancers. The manuscript presents a substantial amount of valuable new knowledge and, as such, I think the paper is suitable for PLos Biol. I have the following suggestions for minor revisions to improve the paper.

RESPONSE:

We thank the reviewer for the positive assessment of the manuscript.

An obvious question is which transcription factor works with Yap in T cells. Genetic experiments with TEADs would be very challenging, given there are 4 but can the authors make predictions on whether TEADs will be relevant here? For example, they could assess TEAD1-4 expression in the different T cell subsets at different stages of development and/or mine their RNA-seq data for expression of TEADs. Although a correlative rather than definitive experiment, it might also be possible to assess the relative presence of TEAD motifs in their RNA-seq datasets.

RESPONSE:

We have added new experimental data analyzing the expression of TEADs following T cell activation in Figure 1G-H. Interestingly, we observed TEAD1 and TEAD3 were upregulated. New unbiased bioinformatic analyses of our RNA-seq data showed significant enrichment of TEAD3 motif-associated genes (new Supplemental Figure 5) in both CD4+ and CD8+ TILs. Additional support for TEAD-dependent mechanisms are provided by our verteporfin experiments (Fig. 1I-J, Supplemental Figure I-J), since verteporfin is a described inhibitor of Yap-TEAD activity.

**Reviewer #2**: The author’s present exciting observations that Yap is highly upregulated in both CD4 and CD8 T cells following stimulation. They then go on to generate a conditional Yap knockout in T cells by crossing Floxed Yap mice with a Cd4-Cre.  They find that the CD4 T cells from these mice have a higher capacity to generate Th1/Th2/Th17 cells and YapKO-CD8 T cells had a higher potential to infiltrate tumors and generate an anti-tumor response. These data are therefore very intriguing and have high potential in anti-tumor immune responses.

RESPONSE:

We thank the reviewer for the positive assessment of the manuscript.

*A few issues should be addressed:*

- This study (as well as a previous study from Ni et al., Cancer Discovery 2018; ref 37) assesses the function of Yap-deficient T cells in a conditional knockout which generates thymic progenitors that are knockout for this gene (using the CD4-Cre). As such, mature T cells in the periphery are generated from Yap-KO double positive thymocytes. The problem is that the authors assume that the selection of WT and Yap-KO thymocytes is equivalent (to be fair, this assumption is also made in the Ni et al publication). As this may not be the case, the data presented may be due to differences in the TCR selection process in the thymus and NOT directly to the lack of Yap in peripheral T cells. This issue is not simple to address but could be evaluated by

1) crossing the conditional YapKO mice to a transgenic TCR and evaluating the ability of those T cells to mount an immune response against a tumor bearing that antigen (i.e. OT1 TCR and B16-Ova tumor);

2) knocking down Yap in peripheral T cells from WT mice by an shRNA approach

3) introducing Yap (i.e. retroviral transduction) into the KO T cells to assess whether the observed differences are inhibited

RESPONSE:

We agree with the reviewer’s concern. To address the question of differential selection directly, we extensively analyzed cell populations in the thymus comparing Yap-cKO to WT mice. If Yap-cKO in DP thymocytes was causing differential selection, we would expect significant shifts in cell number at various maturation stages (i.e. post-DP thymocytes) or in markers of TCR engagement/selection such as Nur77 and CD69. However, as shown in our new Figure 3 and discussed in the new Results section “Yap deletion does not alter T cell development or output in the thymus”, there were no significant changes in thymocyte number at major stages of development (DN, DP, SP; Fig. 3A). We also show no changes in TCRβ positive selection and CD69 expression (Fig. 3B) stages, SP maturation in the medulla among TCRβ+CCR7+ thymocytes (Fig. 3C-E), and found that the sensitive marker Nur77 for negative selection/recent TCR engagement (Fig. 3F) were comparable in Yap-cKO and WT mice.

These data significantly strengthen our story by providing the first evidence that thymic maturation and output from Yap-cKO mice are equivalent to WT cells. We thank the reviewer for enhancing the manuscript with this line of inquiry. The new data also support the hypothesis that Yap in mature T cell function is not due to effects of Yap-cKO on thymocyte maturation, or due to shifts in T cell positive and negative selection processes.

As the authors point out, their data differ from those published by Ni et al. which reported Treg-specific functions for Yap. Given these differences, what are the kinetics of Yap upregulation (and downregulation) and how does it compare to CD25/CD71?

RESPONSE:

Yap is rapidly induced following activation of T cells. As shown in new data (Fig 1A), Yap protein levels peak at 6 h and are sustained at elevated levels 24 h post-activation. New data on activation markers at day 3 post-activation (Figure 1C-F, Supplemental Figure 1E-F) demonstrated unique dose-responsive sensitivity of T cell activation markers CD44, CD25, and CD69, showing Yap-cKO T cells are more sensitive than WT cells.

How does the induction of Yap compare in Treg and Th1 effector conditions? Do the authors here also find a decreased ability of Yap-ko naive CD4 T cells to generate Tregs?

RESPONSE:

In new data presented in Figure 2D, we observed an increase in the sensitivity of Yap-cKO CD4+ T cells to Treg induction by TGFβ. We observed higher percentages of FoxP3+ Yap-cKO CD4+ T cells compared to WT cells, and have discussed this point in the revised manuscript.

Interestingly, we see effects of Yap on early T cell activation markers and rapid induction post-activation. These early changes set the stage for all effector conditions as shown in Figure 2. This observation is supported by the increased effector differentiation under all CD4+ polarizing conditions (Th1, Th2, Th17, Treg) we tested, which suggest that increased YAP levels are a global negative regulator for all T cell differentiation conditions.

As the relative induction of Yap in figure 1 appears to be 70 in CD4 T cells and 45 in CD8 T cells; is it induced to higher levels in CD4s?

RESPONSE:

Our new western blot data (Fig. 1A) show that YAP is induced in both CD4+ and CD8+ T cells by activation, and that protein levels are generally similar. We also analyzed our RNA-seq studies, which show normalized YAP expression in CD4+ and CD8+ tumor-infiltrated T cells are similar in the CD4+ and CD8+ T cells (included below for reviewer reference, Reviewer Fig 1). While there may be potential gene regulatory differences in the timing of YAP expression upon activation, we feel that understanding these differences are beyond the scope of this manuscript.

**Reviewer Fig 1**. Analysis of RNA-seq data from our study shows a similar ratio of YAP counts relative to CD3 counts in CD4+ and CD8+ TILs.

The authors state that phenotype of  “Yap-cKO CD4+ TILs are more skewed towards a Th2 and Treg phenotype compared to WT CD4+ TILs”. How then do the authors explain the improved anti-tumor effect of these cells? Is this in contradiction with the previous publication that finds decreased iTreg generation and function in Yap-cKO T cells?

RESPONSE:

We have added clarification to this point in the Results and Discussion, and in general our data is not contradictory to the previous publication. However, our data do support our conclusions that CD4+ T cells have higher potential to differentiate in response to local cues, which are Th2 and Treg skewing in B16F10 tumors (see PMIDs: 9637498, 20160101, 27185917). Our data do not contradict the prior study by Ni et al, which demonstrates that Yap is necessary for Treg function using a FoxP3Cre conditional Yap knockout model. Our study taken together with this prior study suggests that the Yap-deleted Tregs we observed are dysfunctional. We have also added text in the revised discussion underscoring that we observe increased CD8+ T cell infiltration with Yap deletion, which may be the primary mechanism by which tumor growth is restricted.
